# Extracorporeal liver support techniques: a comparison

**DOI:** 10.1007/s10047-023-01409-9

**Published:** 2023-06-19

**Authors:** Ivano Riva, Antonella Marino, Tino Martino Valetti, Gianmariano Marchesi, Fabrizio Fabretti

**Affiliations:** General Intensive Care Unit, Azienda Socio Sanitaria Territoriale Papa Giovanni XXIII, Bergamo, Piazza OMS, 1, 24127 Bergamo, Italy

**Keywords:** Liver failure, Extracorporeal support, Liver support

## Abstract

ExtraCorporeal Liver Support (ECLS) systems were developed with the aim of supporting the liver in its detoxification function by clearing the blood from hepatic toxic molecules. We conducted a retrospective comparative analysis on patients presenting with liver failure who were treated with different extracorporeal techniques in our intensive care unit to evaluate and compare their detoxification abilities. To verify the effectiveness of the techniques, mass balance (MB) and adsorption per hour were calculated for total bilirubin (TB), direct bilirubin (DB), and bile acids (BA) from the concentrations measured. MB represents the total amount (mg or mcMol) of a molecule removed from a solution and is the only representative parameter to verify the purification effectiveness of one system as it is not affected by the continuous production of the molecules, released in the circulation from the tissues, as it is the case for the reduction rate (RR). The total adsorption per hour is calculated by the ratio between MB and the time duration and shows the adsorption ability in an hour. Our comparative study shows the superior adsorption capability of CytoSorb system regarding TB, DB, and BA, evaluated through the MB and adsorption per hour, in comparison with CPFA, MARS, Prometheus, and PAP. In conclusion, as extracorporeal purification in liver failure could be considered useful for therapeutic purposes, Cytosorb, being more performing than other systems considered, could represent the device of first choice.

**Supplementary Information:**

The online version contains supplementary material available at 10.1007/s10047-023-01409-9.

## Introduction

Hepatic dysfunction is a condition characterized by the impairment of the main liver functions: detoxification, synthesis, and regulation. Alteration in the detoxification function is associated with the inability to metabolize various molecules [1, 2], resulting in their accumulation in the systemic circulation. The consequences are metabolic and biochemical alterations affecting mainly the neurological [3–6] and renal function [7–29], and eventually resulting in secondary multiple organ dysfunction [1]. Among these molecules, inflammatory mediators and hepatic toxins accumulate in the circulation, and the last ones include both water soluble compounds (e.g., ammonia) and hydrophobic ones (e.g., bilirubin, bile acids, hydrophobic amino acids, and endogenous benzodiazepines) which are bound to transport proteins in the plasma, which is mainly albumin [1, 2]. The cytotoxicity of these molecules is well known [3–5], especially their effect on the Central Nervous System (CNS), because of their ability to damage astrocytes and neurons through oxidative stress and apoptosis, disrupting transport of neurotransmitters [4]. Therefore, increased blood and brain levels of these molecules have generally been considered to be the crucial factors in the pathogenesis of Hepatic Encephalopathy (HE) detectable both instrumentally with electroencephalogram (EEG) monitoring, and clinically through one of the available scale such as the West Haven Scale [6]. As stated, renal failure represents one of the main complications in liver dysfunction [7–10] and is caused by many trigger factors as systemic inflammation, renal vasoconstriction with portal hypertension, bacterial infection, or cholemic nephrosis [8–10]. The latter represents an important cause of renal dysfunction due to increased plasma concentrations of bile acids and bilirubin, which seem to present nephrotoxic features and are eventually capable of causing symptoms such as incoercible pruritus [11]. Indeed, in liver dysfunction conditions, biopsies reveal accumulation of intraductal and intracellular bilirubin [8, 9] whose removal by the liver is very slow, if not impossible.

ExtraCorporeal Liver Support (ECLS) systems [1, 12–17] were developed in the past with the aim of supporting the liver in its detoxification function by clearing the blood from hepatic toxic molecules, to bridge the patient either to orthotopic liver transplantation or, possibly, to functional recovery. Extracorporeal liver support is indicated in patients with acute liver failure (ALF) or acute-on-chronic liver failure (ACLF), in presence of primary non function (PNF) after liver transplantation or in case of post-hepatectomy liver failure (PHLF) or in patients with intractable pruritus [27–30]. In recent literature, we can find some evidences that ECLS may reduce mortality and improve HE in patients with ALF and ACLF [31]. The removal of bilirubin has always represented a challenging target for the effectiveness of an extracorporeal liver support technique. Conventional dialysis systems are only capable of removing water soluble compounds, thus not effective in removing the majority of hepatic toxins, since many of these substances are strictly bound to proteins. This has led to the development of extracorporeal adsorption-based therapies, mainly based on plasma-adsorption and albumin dialysis. The molecular adsorbent recirculating system (MARS) [13–15] has been extensively used as a liver support system over the years, as well as other techniques such as fractionated plasma separation and adsorption system (Prometheus) [13, 16], plasma adsorption perfusion (PAP) [17], and coupled plasma filtration adsorption (CPFA) [13, 18, 19], which show contradictory data regarding clinical end-points. Despite biochemical evidence of removal of some hepatic toxins, these systems are technically complex with increasing risks of problems during the treatment, which compromise their effectiveness and duration [1, 19, 20]. As a consequence, simple and effective techniques, especially for protein-bound molecules, have been researched for use in liver failure. As such, CytoSorb is a widely studied adsorption cartridge for cytokine removal, which has also shown, both in vitro and in vivo studies, the ability to remove hepatic toxins [20–28] and protein-bound molecules [20].

Limited direct comparison between the different methods is present in the literature [24–26]. In our clinical experience, we have been able to perform all of the main liver support techniques and have observed significant differences in term of effectiveness and system usability.

For this reason, we performed a retrospective comparative analysis on data collected during the extracorporeal treatments used in our center, in particular MARS, Prometheus, CPFA, PAP, and CytoSorb. The main objective was to assess and compare the detoxification capacity of the systems in term of total bilirubin (TB), direct bilirubin (DT), and total bile acids (BA).

## Materials and methods

### Study design and population

We conducted a retrospective comparative analysis on patients (treated in the years from 2008 to 2019) presenting with liver failure who were treated with different extracorporeal techniques in our intensive care unit to evaluate and compare their detoxification abilities.

A total of 39 patients were analyzed which included 17 patients with CytoSorb (28 treatments), 19 with CPFA (37 treatments), 1 with MARS (3 treatments), 1 with Prometheus (5 treatments), and 1 with PAP (2 treatments).

Considering the limited number of MARS, Prometheus and PAP treatments, and since these were also performed at the beginning of our experience, this retrospective analysis focused mainly on the two major techniques used in our clinical experience, i.e., CytoSorb and CPFA, while a limited comparison was performed for all different types of extracorporeal support. The study was approved by the ethics committee of Bergamo no. 133-22.

### Extracorporeal liver support treatments administered

MARS (Gambro, Sweden) and Prometheus (Fresenius Medical Care, Germany) systems are based on the concept of albumin dialysis. In the MARS treatment blood is dialysed across an albumin-impermeable high-flux membrane with a separate secondary circuit pre-filled with 20% albumin solution, and then perfused through an additional low-flux dialysis and a combination of charcoal and anion-exchange resins in the secondary circuit [12–15]. In contrast, the Prometheus treatment is based on plasma separation across an albumin-permeable filter, perfused through a neutral and an anion-exchange resin, and finally, the blood is dialyzed through a high-flux dialyzer in the principal circuit [12, 13, 16].

PAP using Plasorba BR-350 (Asahi Kasei Medical, Japan) and CPFA (Bellco, Italy) systems are based on the concept of plasma-adsorption. PAP purifies the plasma, separated by a plasmafilter, through an adsorption resin column [17], while the CPFA treats separated plasma that passes through a sorbent adsorption cartridge, and then, whole blood is dialyzed through a high permeability dialyzer in the principal circuit [18].

CytoSorb (CytoSorbents Corp., USA) [20–23] is a sorbent cartridge made of biocompatible polymers for hemoperfusion of the whole blood, which is able to adsorb a broad spectrum of hydrophobic compounds with a molecular weight between 10 and 55 kDa, including inflammatory mediators or, including albumin-bound, hepatic toxins [20]. The system can be easily integrated in combination with commonly used continuous renal replacement systems (CRRT), and does not need dedicated equipment or plasma separation.

For all the treatments, blood, plasma and dialysate flow rate, whereas necessary, were set according to the hemodynamic situation of the patient, and according to the manufacturer’s recommendations.

### Treatment effectiveness

To verify the effectiveness of the treatments, mass balance (MB) and adsorption per hour were calculated for TB, DB, and BA from the concentrations measured.

MB represents the total amount (mg or mcMol) of a molecule removed from a solution and is the only representative parameter to verify the purification effectiveness of one system as it is not affected by the continuous production of the molecules, released in the circulation from the tissues, as is the case for the reduction rate (RR).

In in vivo treatments, concentrations are not available every minute; therefore, MB is calculated by averaging the delta concentrations of two adjacent time levels and multiplying the result by the time and plasma flow (*Q*_plasma_).

The formula is as follows:

$${\text{MB }}\left[ {{\text{mg}}} \right]{ = }\left[ {\left( {C_{0} \left( {t_{0} } \right){-}C_{{\text{f}}} \left( {t_{0} } \right)} \right) + \left( {C_{0} \left( {t_{x} } \right){-}C_{{\text{f}}} \left( {t_{x} } \right)} \right)} \right]/2 \times Q_{{{\text{plasma}}}} \times t_{x} .$$

With *C*_0_ and *C*_f_ being the concentration pre- and post-removal system at every interval *t*_*x*_, respectively.

The total adsorption per hour is calculated by the ratio between MB and the time duration and shows the adsorption ability in an hour.

Reduction rate [RR] was also evaluated from the time course of the systemic toxins levels and is otherwise expressed as the difference between the baseline and final concentrations with the following formula:

$${\text{RR}}\left[ \% \right] = \left[ {\left( {C_{0} \left( {t_{0} } \right) \, {-} \, C_{{\text{f}}} \left( {t_{0} } \right)} \right)/C_{0} \left( {t_{0} } \right)} \right] \times 100.$$

As all these molecules are cleared from the plasma fraction of the whole blood and, to compare all the techniques, plasma flow was considered. In the plasma-adsorption treatments plasma flow was extrapolated from the parameters set-up, otherwise estimated considering the patient hemoconcentration, as follows:

$$Q_{{{\text{plasma}}}} \left[ {\text{ml/min}} \right] = Q_{{{\text{blood}}}} \times \left[ {1 - {\text{hematocrit}}} \right].$$

### Data collection

Serum samples were collected on average every 2 h during the course of the treatments directly from the extracorporeal circuit before and after the adsorbent systems to evaluate the absolute reduction of toxins and to analyze their removal kinetics over time. This choice was made because of the inability of the patient's plasma concentration to give indications about purifying effectiveness due to the continuous production of the molecules. Biochemical measures were performed using standard laboratory procedures.

### Statistical analysis

All the calculations were performed using NCSS Statistical Software, version 10, (NCSS, Kaysville, UT).

All data are presented as mean ± standard error of the mean (SEM) unless indicated otherwise. In case of median, minimum and maximum value (Min, Max) are represented. Comparisons between the different groups were performed using non-parametric methods, as Mann–Whitney Test and Kruskal–Wallis Test for multiple group comparisons, as appropriate. The ANOVA model for repeated measures was applied to test the effects of time, group, and the two-factor interaction. Differences were considered statistically significant at *p* < 0.05.

## Results

### Treatments performance

Technical characteristics of the study treatments are reported in Table 1.

**Table 1:** Technical characteristics of the study treatments

	CYTOSORB	CPFA	MARS	PAP	PROM
Number of patients	17	19	1	1	1
Total treatments number	28	37	3	2	5
Number sessions per patient^a^	1 (1–5)	1 (1–7)	2 (1–3)	1.5 (1–2)	3 (1–5)
Total treatment duration (hours)^a^	22 (7–26)	7 (4–14.5)	6 (4.5–9.5)	4.5 (4–5)	5 (3.75–7.5)
Blood flow ratio (ml/min)^a^	100 (100–120)	100 (100–150)	200 (200–200)	150 (150–150)	180 (180–200)
Plasma flow ratio (ml/h)^a^	4200^b^ (4200–5400)	900 (600–1250)	9000 (9000–9000)	1800 (1800–1800)	21,000 (15,000–21,000)

^a^Data are shown as median (Min, Max)

^b^Value estimated from blood flow and hematocrit

Treatments were well tolerated hemodynamically, and no major procedure-related adverse events occurred. Technical differences were experienced in term of anticoagulation in that only heparin could be used with MARS, Prometheus, PAP, and CPFA, whereas CytoSorb could be performed both with both heparin and regional citrate anticoagulation (RCA), and in terms of set-up of the extracorporeal circuit and management of the treatment.

### Treatment purification efficacy

Detoxification ability comparison between CytoSorb and CPFA is reported in Table 2.

**Table 2:** TB, DB, and BA total MB and reduction/h regarding CytoSorb and CPFA (Mann–Whitney)

		CytoSorb (*n* = 28)	CPFA (*n* = 37)
Total bilirubin (TB)	Total mass balance (mg)	2850.05 ± 384.76***	536.53 ± 33.48
	Total adsorption/h (mg/h)	193.85 ± 51.04*	67.69 ± 4.22
Direct bilirubin (DB)	Total mass balance (mg)	2321.57 ± 349.29***	386.98 ± 26.18
	Total adsorption/h (mg/h)	140.33 ± 31.27*	48.92 ± 3.63
Bile acids (BA)^a^	Total mass balance (mcMol)	1689.44 ± 216.69***	485.34 ± 43.10
	Total adsorption/h (mcMol/h)	81.24 ± 8.96	60.01 ± 6.07
Direct bilirubin (DB)/total bilirubin (TB)	DB/TB (baseline/end) index	1.03 ± 0.01	1.00 ± 0.01

Data are shown as mean ± SEM

^a^Different number of samples CytoSorb (*n* = 27) and CPFA (*n* = 29)

^*^p < 0.05 CytoSorb versus CPFA

^***^p < 0.001 CytoSorb versus CPFA

For all the markers, TB, DB, and BA, the total adsorption obtained with CytoSorb, expressed by the total MB value, was significantly higher compared with that obtained with CPFA (*p* < 0.001); nevertheless, the basal characteristics of the patients were not significantly different between the groups (as shown in supplementary Table ST1). This result was more pronounced for TB: 2850.05 (± 384.76) mg with CytoSorb versus 537.45 (± 33.71) mg with CPFA (*p* < 0.001). The adsorption ability was also confirmed by the average adsorption per hour. CytoSorb presented a significantly higher adsorption ability for TB and DB compared to CPFA (*p* < 0.05), whereas the adsorption of BA per hour did not differ between the two techniques. DB/TB ratio, expressing the ability of adsorbing direct and indirect bilirubin, was stable with both devices; however, the result did not reach statistical significance.

**Fig. 1:**
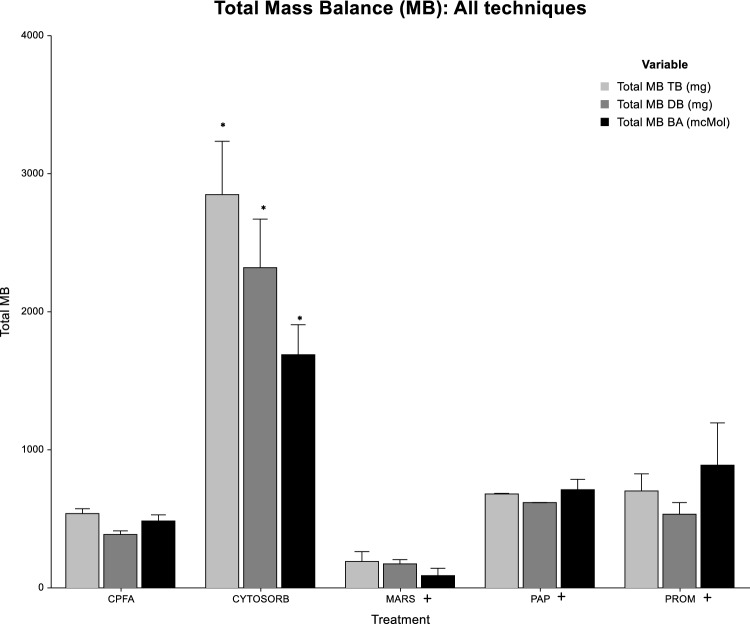
TB, DB, and BA Total MB regarding all the techniques comparison for the overall treatments (Kruskal–Wallis). (Asterisk) Total MB – TB, DB, BA: *p* < 0.05 CYTOSORB vs CPFA, MARS, and PROM; + Limited number of samples PAP (*n* = 2), MARS (*n* = 3), and PROM (*n* = 5)

When performing a comparison between all the techniques (Fig. 1), CytoSorb resulted the most efficient system, showing a total MB value for TB, DB and BA that was significantly higher than CPFA, MARS, and PROM (*p* < 0.05).

### Time course of purification

**Fig. 2:**
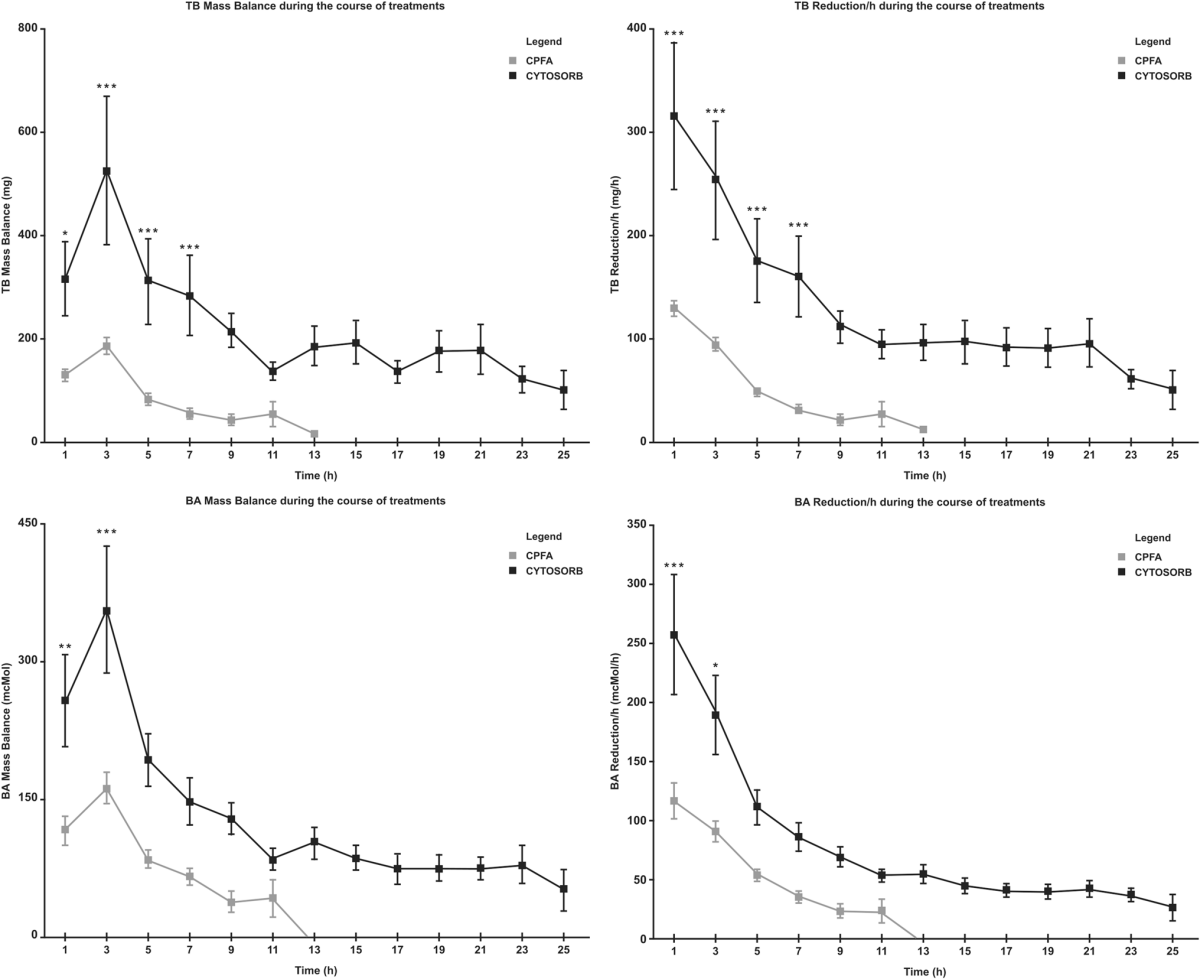
TB and BA MB and reduction/h comparison regarding CYTOSORB versus CPFA during the course of the time (repeated-measures ANOVA). *p < 0.05 CYTOSORB versus CPFA. **p < 0.01 CYTOSORB versus CPFA. ***p < 0.001 CYTOSORB versus CPFA

The evolution of TB, DB, and BA concentrations during the course of the treatments is reported in Table 3. At the beginning, the levels of TB and DB showed a more critical baseline condition in the CytoSorb group, while BA values were comparable among the two groups. The toxin concentrations in the CytoSorb group declined significantly over time up to the end of the treatment (*t* = 25 h); however, in the CPFA group, the reduction was significant only up to 9 h, where we then observed an increase in serum levels. RR did not reach significance. As seen when observing the MB values of TB and BA (Fig. 2), CPFA adsorption ability generally declined toward zero at the end of the treatment, whereas CytoSorb showed continuous adsorption throughout the treatment course, even if it was with less intensity. At all-time points up to 7 h, the difference was significantly higher during CytoSorb treatments for TB and DB, and up to 3 h for BA. This was also visible in the adsorption per hour evolution, which remained higher throughout the duration of CytoSorb treatments (as shown in supplementary figure SF1).

**Table 3:** Time course of pre-cartridge concentrations of the toxins

	Timepoints (h^a^)	1	3	5	7	9	11	13	17	21	25	p**	RR^b^ (%)
TB (mg/dl)	CytoSorb	34.15 ± 4.20 (*n* = 27)	31.82 ± 4.07 (*n* = 27)	30.70 ± 3.96 (*n* = 27)	30.23 ± 3.9 (*n* = 27)	27.52 ± 3.2 (*n* = 25)	28.54 ± 3.36 (*n* = 23)	26.75 ± 3.09 (*n* = 22)	26.91 ± 3.17 (*n* = 22)	26.55 ± 4.27 (*n* = 16)	21.05 ± 3.99 (*n* = 6)	< 0.001 (3–25 h)	38.36
	CPFA	22.26 ± 1.45 (*n* = 35)	19.51 ± 1.31 (*n* = 37)	19.51 ± 1.31 (*n* = 36)	17.82 ± 1.49 (*n* = 28)	18.75 ± 2.35 (*n* = 16)	19.54 ± 4.53 (*n* = 7)	23.3 ± 9.7 (*n* = 3)				< 0.001 (3–9 h)	− 5.14
DB (mg/dl)	CytoSorb	27.97 ± 3.64 (*n* = 26)	24.76 ± 3.26 (*n* = 26)	24.90 ± 3.56 (*n* = 26)	24.42 ± 3.4 (*n* = 26)	22.47 ± 2.97 (*n* = 24)	23.42 ± 3.19 (*n* = 22)	22.48 ± 3.21 (*n* = 21)	22.53 ± 3.17 (*n* = 21)	22.52 ± 4.09 (*n* = 16)	16.92 ± 3.44 (*n* = 6)	< 0.001 (3–25 h)	39,51
	CPFA	17.14 ± 0.99 (*n* = 35)	14.77 ± 0.87 (*n* = 37)	14.77 ± 0.87 (*n* = 36)	13.47 ± 1.08 (*n* = 28)	14.22 ± 1.44 (*n* = 16)	14.46 ± 2.65 (*n* = 7)	16.68 ± 5.56 (*n* = 3)				< 0.001 (3–9 h)	2.68
BA (mcMol/l)	CytoSorb	126.95 ± 16.18 (*n* = 26)	112.59 ± 11.53 (*n* = 26)	106.96 ± 10.96 (*n* = 26)	103.47 ± 10.49 (*n* = 26)	101.47 ± 9.57 (*n* = 25)	102.42 ± 9.71 (*n* = 23)	101.93 ± 9.87 (*n* = 22)	98.24 ± 9.91 (*n* = 22)	106.97 ± 11.67 (*n* = 16)	102.84 ± 19.08 (*n* = 6)	< 0.05 (5–25 h)	18.99
	CPFA	123.33 ± 16.43 (*n* = 27)	91.33 ± 11.43 (*n* = 28)	91.33 ± 11.43 (*n* = 27)	79.27 ± 13.93 (*n* = 20)	82.91 ± 11.05 (*n* = 10)	83.13 ± 10.84 (*n* = 6)	85.87 ± 9.78 (*n* = 2)				< 0.001 (3–9 h)	30.37

^a^h: hours of treatment

^b^RR: removal rate

**Reduction at different time points versus t1h (repeated-measures ANOVA)

## Discussion

In the context of hepatic dysfunction, the removal of cytokines and hydrophobic, albumin‐bound hepatic toxins, such as bilirubin, bile acids, and amino acids, may have a beneficial effect on the clinical course of patients in liver failure [1]. Many extracorporeal liver support systems have been developed over the years, with the focus of removing these accumulating (mainly albumin-bound) toxins from the blood circulation, which has always represented a challenging removal target. To identify the most suitable principle to efficiently support the detoxification liver function, systems mainly based on albumin dialysis and plasma adsorption have been studied, including MARS, Prometheus, CPFA, and PAP [14–19]. More recently, a simple hemoperfusion system, CytoSorb, has demonstrated its ability to modulate inflammatory mediators, as well as bilirubin and bile acid levels [20–22].

Considering the limited comparisons available in the literature [23–25] we performed a retrospective comparative analysis on data collected with different extracorporeal liver support systems in our intensive care unit (Table 1). The objective was to evaluate the detoxification ability regarding TB, DB, and BA.

The main comparison was performed among patients treated with CytoSorb or CPFA due to similar treatment numbers, and the ability of both techniques to significantly adsorb the studied hepatic toxins. However, CytoSorb showed a significantly higher capacity expressed in term of MB. As shown in Table 2, the CytoSorb TB MB is five times more elevated than CPFA and three times for BA. This difference is confirmed when observing the evolution of MB over the treatment time (Fig. 2), and is also noticeable when considering adsorption in the first three hours of treatment, therefore giving a comparable treatment time among the two techniques. Indeed, TB MB was significantly higher for CytoSorb than CPFA (525.85 ± 35.84 vs 185.59 ± 30.62, *p* < 0.001), and so was BA MB (356.08 ± 25.62 vs 162.01 ± 24.69, *p* < 0.001).

The purification effectiveness was maintained by the CytoSorb device throughout the treatment duration, even if with less intensity toward the end and, importantly, no bilirubin release was observed. This was confirmed earlier in an in vitro study published by Gemelli et al. [20]. On the other hand, CPFA purification effectiveness tended toward zero at the end of treatment. Evaluation of the RR (Table 3) did not show significant results which is explained when considering the inability of this parameter to explain the effectiveness of an adsorption device. Indeed, MB is the only representative value to verify the purification effectiveness of one system as it is not affected by the continuous production of the molecules and ongoing release from the tissues, as is the case for RR.

Considering also the other techniques (Fig. 1)—even if the comparison is limited by treatment numbers—these results are confirmed, and CytoSorb showed the greater performance in term of MB (*p* < 0.05).

First of all, this higher capability might be explained by the elevated and available CytoSorb adsorption surface at the beginning of the treatment. Second, the different treatment durations certainly affect the total adsorption ability of the systems: CytoSorb is a system able to work up to 24 h, and in our experience, the median duration was 22 h (7, 26), whereas CPFA treatment was shorter because of technical and saturation issue, so that the median duration was 7 h (4, 14.5). This was the same for the other techniques which were shorter than the CytoSorb treatment (Table 1): MARS 6 h (4.5, 9.5), PAP 4.5 h (4, 5), and PROM 5 h (3.75, 7.5).

Other factors that could affect CytoSorb’s superior removal ability were the elevated TB and BA baseline concentrations, but this was valid for all adsorption therapies in general. The adsorption capacity of the CytoSorb cartridge is clearly dependent on the concentration of the target molecules, as it works in a concentration-dependent manner, efficiently removing high concentrations of target molecules with the goal of modulating the excess levels of toxic molecules, to regain control in complex situations. Remarkably, notwithstanding the higher baseline values, CytoSorb was able to significantly reduce TB, DB, and BA right up to the end of the treatment, reaching similar levels to that of CPFA (Table 3).

It is important to underline that both CytoSorb and CPFA seemed to be able to adsorb unconjugated bilirubin—a strongly albumin-bound molecule—together with direct bilirubin. This has already been demonstrated for CytoSorb [20] and is reiterated in our study considering the stability of the DB/TB ratio. This remained constant between baseline and end of the treatment values, confirmed by the DB/TB index around 1 (Table 2). Indeed, if more direct than unconjugated bilirubin were adsorbed, this index would have been different at baseline and at end of the treatment.

The ability to adsorb albumin-bound toxins might also explain the BA removal. BA are albumin-bound hepatic toxins, even if less tightly bound to albumin than bilirubin [25]. Unconjugated bilirubin presents an affinity binding of 9.5 × 10^7^ M^−1^, unlike the two primary bile acids, cholic acid (CA) and chenodeoxycholic acid (CDCA), 5.5 × 10^4^ M^−1^ and 0.3 × 10^4^ M^−1^, respectively. The different composition of BA—and its affinity binding—may affect the removal efficiency of the systems, which are made from hydrophobic resin, and this could explain the minor adsorption obtained compared to the one for TB, even if we were not able to discriminate among the two BA types and understand their behavior. Nevertheless, the total BA adsorption was significantly superior with CytoSorb compared to CPFA (Table 2).

Technically, the experience with MARS, Prometheus, PAP, and CPFA underlines the need for careful management of anticoagulation, mainly heparin, to avoid clotting problems which affect the continuity of the treatment. One advantage of the CytoSorb system is its integration into the normal clinical practice, allowing also RCA. Indeed, the use of RCA guarantees excellent anticoagulation of both the entire extracorporeal circuit and the adsorbent system, maintaining its purifying effectiveness. Considering these benefits, the use of RCA during hepatic insufficiency could be used providing adequate precautions are taken to avoid the risk of citrate accumulation [26]. For example, there should be precautions taken regarding the limitation of blood flow, the use of a cut-off of ionized calcium in the extracorporeal circuit (at least up to 0.4 mMol/l), close monitoring of the total/ionized calcium ratio (which should not exceed the value of 2), and monitoring the values of pH, bicarbonates and lactates, whose increase must lead to the suspension of the RCA infusion by switching to another anticoagulation method.

Furthermore, CytoSorb can be easily inserted into an extracorporeal circuit for CRRT without changing the usual clinical routine, an important point, as renal failure is a frequent complication of liver failure. Therefore, the simplicity of CytoSorb use positively influences the continuity and duration of the treatment, not least the fact there are fewer complications in the set-up phase.

This study has some limitations. First of all, its retrospective nature and the relatively small and variable number of treatments and measurements per patient. However, considering the limited comparisons that include all the main extracorporeal liver systems noted in the literature, we considered it important to report our experience. Moreover, this study was focused on the analysis of the techniques effectiveness and not designed for clinical outcome evaluation. Further investigations are ongoing for this purpose.

## Conclusions

As reported in other studies [24], the high mortality associated with liver failure has been attributed to the accumulation of several metabolites, such as bile acids and bilirubin. These metabolites, normally removed by the liver, would lead to the dysfunction for example of the brain (hepatic encephalopathy), kidney (hepatic-renal syndrome), and, eventually, to death. Indeed, higher TB, DB, and BA removal seems to be an advantage to improve patients’ outcomes. Our comparative study shows the superior adsorption capability of CytoSorb system regarding TB, DB, and BA, evaluated through the MB and adsorption per hour.

From our experience, CytoSorb might represent the most suitable option as a liver support technique, considering the combination of hepatic toxins, cytokine adsorption ability, and technical versatility, as it can easily be inserted in a CRRT circuit.

### Supplementary Information

Below is the link to the electronic supplementary material.

Supplementary file1 (DOC 121 KB)

## Data Availability

Due to the sensitive nature of the research, supporting data is not available to be shared publicly.
