# Supplementary material for: Extracorporeal liver support techniques: a comparison
Source: J Artif Organs. 2023 Jun 19;27(3):261–8. doi: 10.1007/s10047-023-01409-9 (PMC11345327; doi:10.1007/s10047-023-01409-9)

Supplementary Table ST1: basal characteristics of patients

|  | **Cytosorb ^1^** | **CPFA ^1^** | **p (Cytosorb Vs CPFA)** |
| --- | --- | --- | --- |
| **M/F** | 13/4 | 16/3 | NS |
| **Age** | 55.43 (36.00 - 79.00) | 53.19 (34.00 - 67.00) | NS |
| **ALF*/ACLF*/OTHER** | 10/7/- | 5/9/5 | NS |
| **T BILIRUBIN (mg/dl)** | 30.94 (13.60 - 72.50) | 32.34 (14.70 - 45.70) | NS |
| **D BILIRUBIN (mg/dl)** | 25.02 (8.60 - 56.60) | 23.19 (10.20 - 33.60) | NS |
| **BILE ACIDS (mcmol/L)** | 129.55 (16.40 - 405.00) | 131.70 (26.6 - 379.30) | NS |
| **AST (U/L)** | 490.71 (23.00 - 3024.00) | 137.93 (35.00 - 431.00) | NS |
| **ALT (U/L)** | 275.95 (22.00 - 2062.00) | 71.43 (6.00 - 168.00) | P < 0,05 |
| **PT-INR** | 2.14 (1.25 - 8.18) | 2.25 (1.14 - 4.95) | NS |
| **aPTT** | 2.11 (1.07 - 7.55) | 1.98 (1.25 - 4.45) | NS |
| **CREATININE (mg/dl)** | 1.97 (0.42 - 5.46) | 2.16 (0.80 - 5.64) | NS |
| **UREA (mg/dl)** | 133.38 (25.00 - 312.00) | 113.50 (46.00 - 269.00) | NS |
| **ALBUMIN (mg/dl)** | 3308.45 (2366.00 - 4828.00) | 3506.31 (2790.00 - 4594.00) | NS |
| **AMMONIA (micmol/l)** | 69.05 (18.00 - 146.00) | 62.85 (6.00 - 178.00) | NS |

^1^Data are shown as median (Min - Max)

*According to EASL Guidelines: European Association for the Study of the Liver. Electronic address: easloffice@easloffice.eu; Clinical practice guidelines panel; Wendon, J; Panel members; Cordoba J, Dhawan A, Larsen FS, Manns M, Samuel D, Simpson KJ, Yaron I; EASL Governing Board representative; Bernardi M. EASL Clinical Practical Guidelines on the management of acute (fulminant) liver failure. J Hepatol. 2017 May;66(5):1047-1081. doi: 10.1016/j.jhep.2016.12.003. PMID: 28417882.

Supplementary figure SF1: adsorption per hour (first 3 hours average)

a)


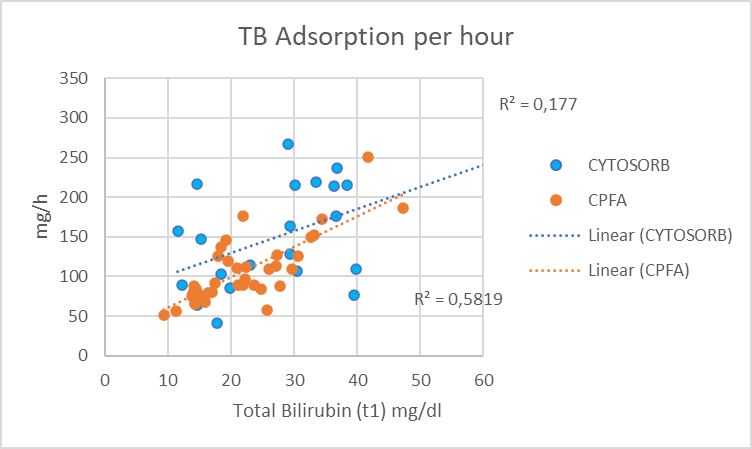


b)


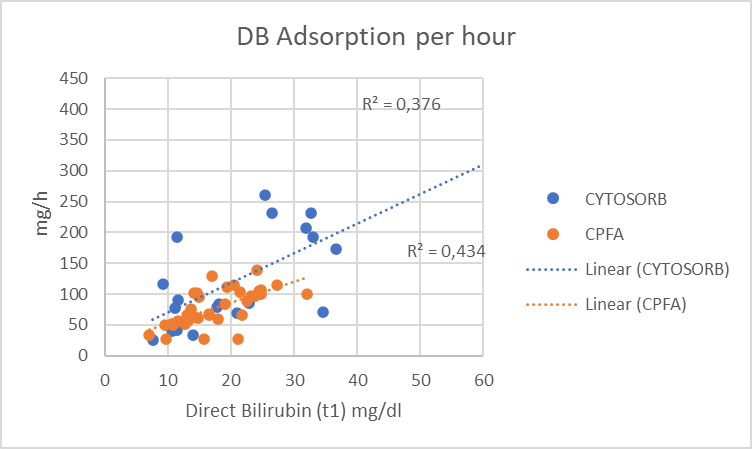


c)


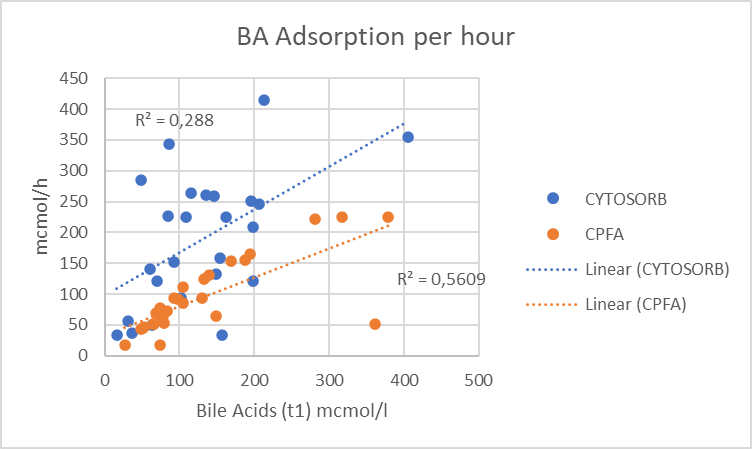

Supplement: Supplementary file 1 — Supplementary file1 (DOC 121 KB) [file 10047_2023_1409_MOESM1_ESM.doc]
